# Associations of anxiety and depression with sexual health among infertility patients: a systematic review

**DOI:** 10.3389/fpsyg.2026.1823084

**Published:** 2026-08-28

**Authors:** Changmin Niu, Yuxin Gao, Qiulian Xu, Fan Yang, Shikun Ma, Nuo Shen, Wenxuan Zhu, Yidi Kong, Lu Chen, Guiqin Wang

**Affiliations:** 1 Department of Gynecology and Obstetrics, Affiliated Hospital of Yangzhou University, Yangzhou, Jiangsu, China; 2 School of Nursing, Faculty of Medicine, Yangzhou University, Yangzhou, China; 3 School of Basic Medical Sciences & School of Public Health, Faculty of Medicine, Yangzhou University, Yangzhou, China; 4 Yangzhou Hospital of Traditional Chinese Medicine, Yangzhou, Jiangsu, China; 5 Department of Anesthesiology, Affiliated Hospital of Yangzhou University, Yangzhou, Jiangsu, China

**Keywords:** anxiety, depression, infertility, sexual health, systematic review

## Abstract

**Purpose:**

This study aims to systematically review the associations between anxiety, depression, and the sexual health of patients with infertility, providing a theoretical foundation for reproductive health professionals to develop targeted interventions.

**Methods:**

A comprehensive literature search was conducted in four English databases (Cochrane Library, EMBASE, Web of Science, and PubMed) and two Chinese databases (CNKI and Wan Fang) from inception to December 31, 2025. Studies were selected following the Preferred Reporting Items for Systematic Reviews and Meta-Analyses (PRISMA) guidelines, and the quality of the included studies was assessed using the Joanna Briggs Institute Critical Appraisal Checklist for Case-Control Studies and the Joanna Briggs Institute Checklist for Analytical Cross-Sectional Studies, as appropriate. All included studies were observational. A meta-analysis was not performed because of substantial heterogeneity in the characteristics of the included studies.

**Results:**

A total of 21 studies (*n* = 6,837 participants) were included, with sample sizes ranging from 50 to 1,468. The findings indicated that anxiety and depression were associated with poorer sexual health among patients with infertility. In women with infertility, higher levels of anxiety and depression were associated with lower libido and sexual arousal, greater difficulties with lubrication and orgasm, and more sexual pain. In men with infertility, anxiety and depression were associated with lower libido, greater difficulty experiencing orgasm, premature ejaculation, and poorer erectile function. Anxiety and depression were also associated with poorer intimacy and communication between partners, including lower perceived security and self-esteem.

**Conclusion:**

These findings support consideration of psychological and sexual health assessments as components of routine infertility care. Family and partner support may also be considered within comprehensive care; however, its effects on physical, psychological, relationship, and quality-of-life outcomes require evaluation in prospective or interventional studies.

## 1 Introduction

Infertility is considered to affect millions of individuals and couples worldwide, often resulting in devastating social and health consequences, including social stigma, financial hardship, as well as physical and mental health problems (Thoma et al., 2021). Approximately one out of every six couples of childbearing age worldwide were affected by infertility, with rates ranging from 12.6% to 17.5% (Cox et al., 2022). Couples often face psychological issues such as depression, anxiety, and problems adapting to married life (Monga et al., 2004; Kiani et al., 2020).

Sexual health is defined as “a state of physical, emotional, mental, and social wellbeing concerning sexuality” (Mediå et al., 2023). The International Classification of Functioning, Disability, and Health (ICF) categorizes sexual health into two areas: sexual function and intimacy. Intimacy, a component of sexual health, encompasses emotional closeness, trust, and the quality of partner communication, with self-disclosure and perceived partner responsiveness serving as core interpersonal processes (Bergeron et al., 2024; Perrier Léonard et al., 2025). For patients with infertility, impairment of sexual health is manifested not only as sexual dysfunction but also as a decline in the quality of intimate relationships and partner interactions, and these changes may further affect overall quality of life and psychosocial adaptation. Previous studies have shown that patients with infertility have a higher incidence of sexual dysfunction compared to those who are fertile (Shahraki et al., 2019). Researchers have proved that the percentage of women with infertility experiencing sexual dysfunction ranges from 11% to 93% (Drosdzol and Skrzypulec, 2008; Khademi et al., 2008; Bayar et al., 2014; Yeoh et al., 2014), premature ejaculation and erectile dysfunction are also relatively common among males (Purcell-Lévesque et al., 2019), with prevalence ranging from 18% to 62% (Drosdzol and Skrzypulec, 2008; Khademi et al., 2008). Furthermore, a decline in sexual quality of life may further weaken the intimate relationship between couples and exacerbate issues such as shame, social suffering, and insufficient social support. Accumulating evidence suggests that anxiety and depression play important roles in the impairment of sexual health among patients with infertility. Previous studies have found that depression significantly impairs male sexual functions (Araujo et al., 1998). Similar phenomena have been observed in patients with infertility, where emotional distress and anxiety caused by infertility may induce or exacerbate sexual dysfunction, which in turn may further impede normal sexual intercourse and affect reproductive outcomes (Mansour et al., 2026). Therefore, a complex bidirectional relationship may exist among anxiety, depression, and sexual health. While psychological factors such as anxiety and depression significantly impact sexual health in patients with infertility, it is imperative to first rule out organic causes (Sahin et al., 2017).

While several scholars have explored topics related to sexual health in patients with infertility, previous reviews and related studies have predominantly been confined to overall sexual dysfunction, as evidenced by studies (McCabe et al., 2010; Kucur Suna et al., 2016; Lotti and Maggi, 2018; Winter et al., 2022). Notably, these previous investigations did not systematically examine the associations of anxiety and depression with the different dimensions of sexual health, particularly intimacy-related outcomes, among women, men, and couples experiencing infertility. In contrast, the present study offers a multidimensional analysis of how anxiety and depression impact the sexual health of patients with infertility, including both sexual function and intimacy-related outcomes. This systematic review aimed to furnish reproductive health professionals with a more robust theoretical foundation and practical insights, enabling them to devise highly targeted intervention strategies.

## 2 Methods

### 2.1 Aims

This article aims to provide supporting evidence and summarize the impact of anxiety and depression on the sexual health of patients with infertility. The protocol of this systematic review has been registered in the PROSPERO database (CRD 42023407173). The review was initially restricted to cross-sectional studies examining sexual function, with no language restrictions. Following an exploratory assessment of the available literature and discussion among the review team, the eligible study designs were expanded to include relevant case–control studies because they investigated comparable populations, psychological exposures, and sexual health outcomes. The outcome framework was also broadened from sexual function to sexual health to encompass intimacy-related outcomes, including relationship satisfaction, emotional intimacy, dyadic functioning, and partner communication. Owing to the language resources available to the review team, the final eligibility criteria were limited to studies published in English or Chinese. These adjustments aimed to better reflect the diverse domains of sexual health among individuals and couples experiencing infertility, thereby enhancing the comprehensiveness and clinical relevance of the review findings. Following these amendments, all outcomes specified in the amended review plan were reported in the present review. This review followed the PRISMA guidelines to select articles.

### 2.2 Search strategy

To ensure comprehensive identification of relevant studies, two researchers systematically searched the Cochrane Library, Embase, Web of Science, PubMed, China National Knowledge Infrastructure (CNKI), and Wanfang Data from database inception to December 31, 2025. To enhance the comprehensiveness and accuracy of the search, the strategies for all databases were developed around the same core concepts using a combination of controlled vocabulary terms and free-text terms, with database-specific modifications made as appropriate. The principal search terms included “depression,” “anxiety,” “mood disorder,” “psychological distress,” “infertility,” “orgasm,” “sexual dysfunction, physiological,” “sexuality,” and “sexual behavior.” Boolean operators (AND, OR) and truncation symbols were used to optimize the combinations of search terms and improve search sensitivity and precision. As the core search concepts were consistent across the databases, the complete search strategy is presented for PubMed as a representative example (see Table 1 for details).

**Table 1:** Search strategy of the representative database PubMed.

Number	Search terms
#1	(“Depression”[Mesh] OR “Anxiety”[Mesh] OR “Mood Disorders”[Mesh] OR “Psychological Distress”[Mesh] OR Depressive Symptoms[Title/Abstract] OR Depressive Symptom[Title/Abstract] OR Symptom, Depressive[Title/Abstract] OR Emotional Depression[Title/Abstract] OR Depression, Emotional[Title/Abstract] OR Angst[Title/Abstract] OR Nervousness[Title/Abstract] OR Hypervigilance[Title/Abstract] OR Social Anxiety[Title/Abstract] OR Anxieties, Social[Title/Abstract] OR Anxiety, Social[Title/Abstract] OR Social Anxieties[Title/Abstract] OR Anxiousness[Title/Abstract] OR Disorder, Mood[Title/Abstract] OR Disorders, Mood[Title/Abstract] OR Mood Disorder[Title/Abstract] OR Affective Disorders[Title/Abstract] OR Affective Disorder[Title/Abstract] OR Disorder, Affective[Title/Abstract] OR Disorders, Affective[Title/Abstract] OR Distress, Psychological[Title/Abstract] OR Emotional Distress[Title/Abstract] OR Distress, Emotional[Title/Abstract])
#2	(“Infertility”[Mesh] OR Sterility, Reproductive[Title/Abstract] OR Reproductive Sterility[Title/Abstract] OR Sterility[Title/Abstract] OR Subfertility[Title/Abstract] OR Sub-Fertility[Title/Abstract])
#3	(“Orgasm”[Mesh] OR “Sexual Dysfunction, Physiological”[Mesh]) OR “Sexuality”[Mesh] OR “Sexual Behavior”[Mesh] OR Orgasms[Title/Abstract] OR Sexual Satisfaction[Title/Abstract] OR Satisfaction, Sexual[Title/Abstract] OR Sexual Satisfactions[Title/Abstract] OR Sexual Gratification[Title/Abstract] OR Gratification, Sexual[Title/Abstract] OR Sexual Gratifications[Title/Abstract] OR Physiological Sexual Dysfunction[Title/Abstract] OR Physiological Sexual Dysfunctions[Title/Abstract] OR Sex Disorders[Title/Abstract] OR Sexual Disorders, Physiological[Title/Abstract] OR Physiological Sexual Disorder[Title/Abstract] OR Physiological Sexual Disorders[Title/Abstract] OR Sexual Disorder, Physiological[Title/Abstract] OR Sexual Dysfunctions, Physiological[Title/Abstract] OR Sexual Behavior[Title/Abstract] OR Behavior, Sexual[Title/Abstract] OR Sex Behavior[Title/Abstract] OR Behavior, Sex[Title/Abstract] OR Sexual Activity[Title/Abstract] OR Activities, Sexual[Title/Abstract] OR Activity, Sexual[Title/Abstract] OR Sexual Activities[Title/Abstract] OR Premarital Sex Behavior[Title/Abstract] OR Behavior, Premarital Sex[Title/Abstract] OR Sexual Orientation[Title/Abstract] OR Orientation, Sexual[Title/Abstract] OR Sex Orientation[Title/Abstract] OR Anal Sex[Title/Abstract] OR Sex, Anal[Title/Abstract] OR Oral Sex[Title/Abstract] OR Sex, Oral[Title/Abstract] OR sexual problems[Title/Abstract]
#4	#1 AND #2 AND #3

### 2.3 Inclusion and exclusion criteria

The eligibility criteria were developed according to the Population, Exposure, Outcomes, and Study Design framework. The search strategy was formulated using a combination of Medical Subject Headings and relevant free-text keywords. The inclusion criteria for studies investigating the effects of anxiety and depression on sexual health among patients with infertility were as follows:

- Population: women, men, or couples diagnosed with infertility, regardless of whether infertility was primary or secondary and regardless of the identified cause of infertility.
- Exposure: anxiety and/or depression, assessed using validated self-report questionnaires, clinician-administered instruments, or clinical diagnostic criteria.
- Outcomes: sexual health outcomes, including sexual function and intimacy-related outcomes. Sexual function outcomes included sexual desire, arousal, lubrication, orgasm, sexual satisfaction, sexual pain, erectile function, and ejaculatory function. Intimacy-related outcomes included relationship or marital satisfaction, emotional closeness, dyadic functioning, and partner communication.
- Study designs: observational studies, specifically cross-sectional and case–control studies.

Studies were excluded if the full text or essential information was unavailable; if they were meta-analyses, systematic reviews, case reports, animal studies, or gray literature, including dissertations, conference abstracts, or conference proceedings; if they were published in languages other than English or Chinese.

### 2.4 Data extraction

The studies collected relevant data on subjects, including gender, age, and infertility characteristics (primary infertility, secondary infertility, cause of infertility [female factor, male factor, both, or unexplained]). We also gathered general information, such as author, publication date, country, type of study, research tools, research results, etc. Two reviewers extracted the required information and collaborated to create the first draft of the chart. If there were differing opinions, they were resolved through discussion or by consulting independent third-party researchers (see Table 2 for details).

**Table 2:** Characteristics of included studies (*n* = 21).

Source	Country	Design	Subject	Instruments	Important findings
Jenabi et al., 2025	Iran	cross-sectional study	240 infertility women	DASS-21, FSFI	Depression, anxiety, and stress exhibit strong inter-correlations and robust inverse correlations with all sexual function domains.
Fernandes et al., 2023	Portugal	cross-sectional study	63 pairs of infertility couples, 44 pairs of fertile couples	HADS, IIEF, FSFI	The relationship between depression and sexual function was stronger in infertile men than in men with childbearing potential.
Naumova et al., 2021	Spain	cross-sectional study	37 PCOS infertile women, 36 cases of tubal causes, 36 male causes	FSFI, BDI, HAM-A	There was a significant correlation between the total score of FSFI and the impairment of psychological and emotional states.
Pasha et al., 2021	Iran	cross-sectional study	424 infertile women	FSFI, GHQvn	Depression symptoms were significant predictors of perceived sexual dysfunction.
Ho et al., 2020	Vietnam	cross-sectional study	255 pairs of infertile couples	FSFI, PEDT, IIEF-15, DASS-21	Psychological disorders in both husbands and wives were associated with sexual dysfunction.
Shahraki et al., 2019	Iran	cross-sectional study	115 primary infertility women, 74 secondary infertility women	BDI, BAI, FSFI	There was a significant correlation between the BDI score and the FSFI score.
Coward et al., 2019	United States	cross-sectional study	708 unexplained infertility males	Ferti-QOL, PHQ-9, IIEF	Depressed men were more likely to experience ED than non-depressed men.
Cao et al., 2019	China	cross-sectional study	480 infertile males	HADS, PEDT, IIEF-5	Depression was associated with ED.
Ozturk et al., 2019	Turkey	cross-sectional study	96 infertility women, 96 fertile women	BDI, IFSF	Sexual function decreases when depressive symptoms increase in infertile and fertile women.
Purcell-Lévesque et al., 2019	Canada	cross-sectional study	88 infertile women, 45 pairs of infertile couples	ASEX, Experiences in Close Relationships	Anxiety related to sexual dysfunction.
Shahraki et al., 2018	Iran	cross-sectional study	78 primary infertility women, 71 secondary infertility women, 115 fertile women	BDI, FSFIS, SQOL-F	BDI scores were significantly higher in cases of sexual dysfunction.
Salomão et al., 2018	Brazil	case-control study	140 infertility women, 140 fertile women	FSFI, HADS	Anxiety and depression increased the risk of sexual dysfunction in the study population.
Sahin et al., 2017	Turkey	cross-sectional study	39 primary sterility, 31 secondary sterility	IIEF, BDI	Depression and low income were independent predictors of sexual dysfunction.
Kucur Suna et al., 2016	Turkey	cross-sectional study	Infertility women of different causes: A: 30 female reasons, B: 31 male causes, C: 81 unknown causes	FSFI, BDI	FSFI total score and all FSFI subgroup scores were negatively correlated with BDI score.
Czyżkowska et al., 2016	Poland	cross-sectional study	50 infertility women, 50 fertile women	SSS, BDI, Mell-Krat, FAM-II	BDI was a predictor of SSS in the control group.
Yangin et al., 2016	Turkey	cross-sectional study	102 pairs of infertile couples	BDI, GRISS	A correlation has been observed between the total scores of GRISS and BDI assessments for both men and women.
Gao et al., 2013	China	cross-sectional study	1468 infertile males and 942 healthy male volunteers	PEDT, IELT, IIEF-5, SAS, SDS	Depression was associated with PE or ED.
Pakpour et al., 2012	Iran	cross-sectional study	604 infertility women	IV-FSFI, HRQL, HADS	Risk factors for FSD identified were older age and self-reported depression.
Furukawa et al., 2012	United States	case-control study	75 infertility women, 210 fertile women	FSFI, PHQ-9	There was no difference in FSFI and PHQ-9 scores between the two groups.
Lotti et al., 2012	Italy	cross-sectional study	244 infertile males	IIEF-15-EFD, PEDT, MHQ, NIH-CPSI, SIL-8, CDU	ED was mainly associated with depressive symptoms.
Nelson et al., 2008	United States	cross-sectional study	121 pairs of infertile couples	FSFI, SEAR, IIEF, CES-D, SF-36	There was a tendency to link FSFI scores to depressive symptoms.

PE, premature ejaculation; ED, erectile dysfunction; BDI, beck depression inventory; GRISS, golombok-rust inventory of sexual satisfaction; SSS, the sexual satisfaction scale; mell-krat scale(the version for women):sexual needs and reactions; FAM-III, family assessment measure; FSFI, female sexual function index; HADS, hospital anxiety and depression scale; ASEX, The Arizona sexual experience scale; IFSF, index of female sexual function; FSFIS, female sexual function index; SQOL-F, sexual quality of life-female; PHQ-9, patient-health questionnaire-9; IV-FSFI, Iranian version of the female function index; HRQL, health-related quality of life; PEDT, PE diagnostic tool; IELT, the intravaginal ejaculatory latency time; IIEF-5, international index of erectile function; SAS, self-rating anxiety scale; SDS, self-rating depression scale; IIEF-15-EFD, international index of erectile function-15 erectile function domain; MHQ, middlesex hospital questionnaire; NIH-CPSI, national institutes of health-chronic prostatitis symptom index; SIL-8, hormonal, seminal, and interleukin 8; CDU, scrotal and transrectal color doppler ultrasound; Ferti-QOL, fertility-related QOL; IIEF-15, international index of erectile function 15; DASS-21, depression anxiety stress scales 21; BAI, beck anxiety inventory; SEAR, self-esteem and relationship questionnaire; CES-D, Center for epidemiological studies depression scale; SF-36, short-form-36 for general quality of life; GHQ, general health questionnaire; HAM-A, the hamilton anxiety rating scale.

### 2.5 Data synthesis

Heterogeneity was assessed narratively by comparing studies in terms of study design, participant characteristics, infertility-related characteristics, sexual health outcome domains, measurement instruments, and the direction of findings. This assessment indicated substantial clinical, methodological, and outcome heterogeneity across the evidence base. Therefore, meta-analysis was not considered appropriate. The findings were synthesized narratively and organized according to sexual health outcome domains to provide a structured and rigorous synthesis of the available evidence.

### 2.6 Quality appraisal

To assess the methodological quality of the included studies, two reviewers independently assessed methodological quality using critical appraisal tools appropriate to each study design. Analytical cross-sectional studies were evaluated using the eight-item Joanna Briggs Institute (JBI). Critical Appraisal Checklist for Analytical Cross-Sectional Studies. Each item was scored dichotomously (“yes” = 1; “no” or “unclear” = 0), yielding a total score ranging from 0 to 8. Similarly, case–control studies were assessed using the JBI Critical Appraisal Checklist for Case–Control Studies. Each item was scored using the same dichotomous system, resulting in a possible total score ranging from 0 to 10. No categorical cut-offs were applied because the JBI checklists do not prescribe validated numerical thresholds for classifying overall methodological quality. The assessments were conducted independently by two reviewers trained in evidence-based medicine. Any disagreements were resolved through discussion or, when necessary, arbitration by a third senior reviewer. Formal inter-reviewer agreement statistics were not calculated because the reviewers' original pre-consensus decisions were not available in a form that permitted reliable retrospective calculation. A formal GRADE assessment was not undertaken because substantial clinical and methodological heterogeneity and the narrative nature of the synthesis limited the interpretability of outcome-specific certainty ratings. Confidence in the evidence was therefore discussed narratively, considering study design, risk of bias, confounding, and consistency across studies.

## 3 Results

### 3.1 Literature search results

**Figure 1:**
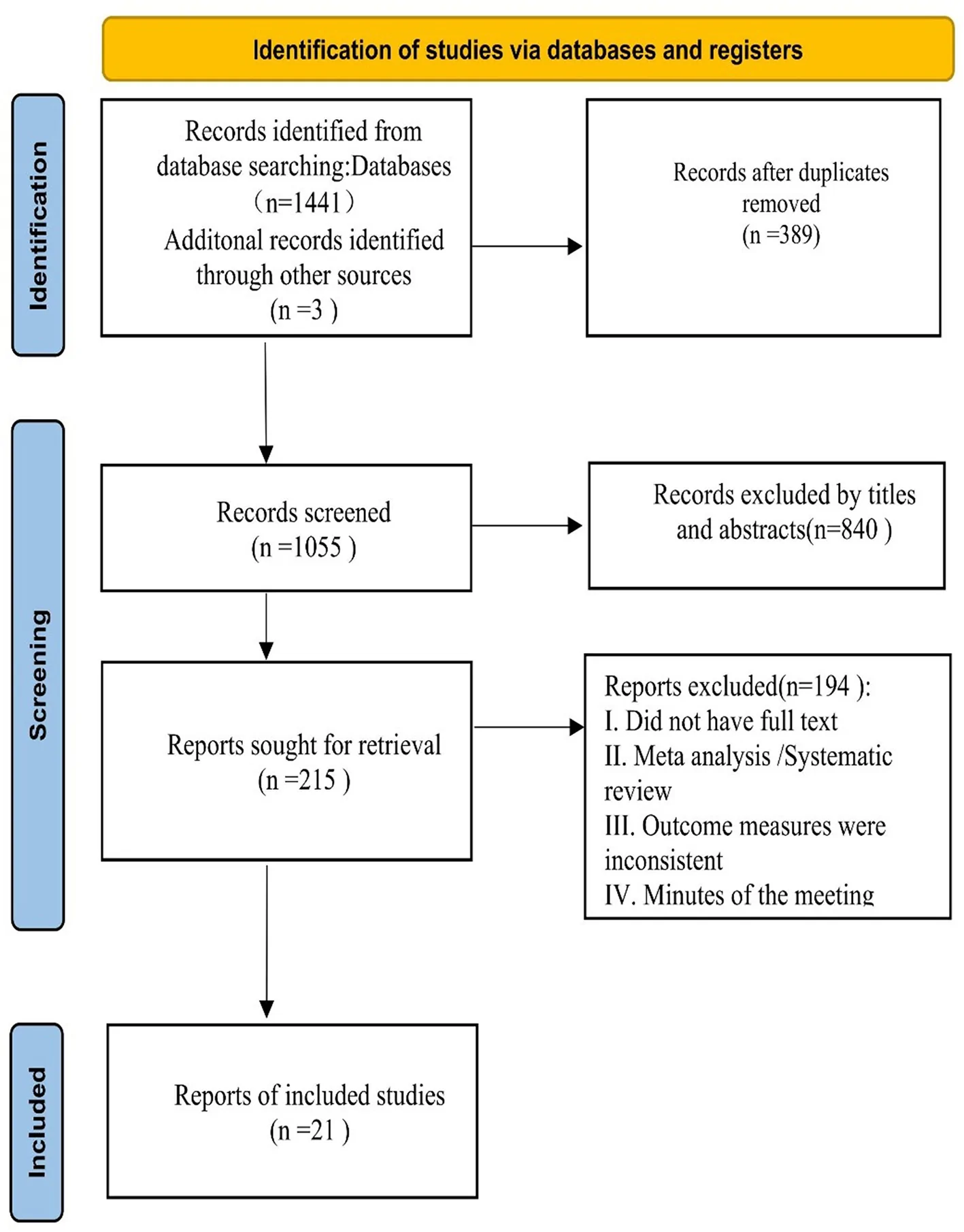
PRISMA flow diagram.

The initial search yielded 1,444 articles. The PRISMA flowchart shown in Figure 1 summarizes the screening process. These records comprised 266 from PubMed, 409 from Web of Science, 456 from Embase, 77 from Cochrane, 45 from CNKI, 188 from the Wanfang Database, and 3 obtained through other sources. After deduplication using Endnote software, 1,055 records remained. Title and abstract screening excluded 840 irrelevant articles. Full-text screening was performed on the remaining 215 articles, of which 194 were excluded for various reasons (such as commentaries, reviews, conference proceedings, etc.). Ultimately, 21 studies were included in the systematic review, involving a total of 6,837 patients. These studies consisted of 2 case–control studies and 19 cross-sectional studies. The study selection process is summarized in Figure 1.

### 3.2 Study characteristics

The fundamental features of the included literature are summarized in Table 2. Study designs included two case–control studies and nineteen cross-sectional descriptive studies. The discussion focused on the associations of anxiety and depression with sexual health among women and men with infertility. Populations involved in this study came from 11 countries with sample sizes ranging from 50 to 1,468 participants per study. Studies were conducted in Turkey (4), the USA (3), Iran (5), China (2), and one each in Portugal, Spain, Poland, Brazil, Canada, Italy, and Vietnam. All included articles have used valid questionnaires.

### 3.3 Methodological quality and risk of bias

Table 3 presents the methodological quality scores of the included studies. Scores ranged from 5 to 8 for the cross-sectional studies and from 5 to 8 for the case–control studies. Across the 21 included studies, the mean percentage quality score was 82.38%. Among the cross-sectional studies, relatively low scores were observed for the use of objective and standardized criteria to define the condition under investigation and for strategies used to identify and address confounding factors. In contrast, higher scores were obtained for the validity and reliability of exposure and outcome measurements and for the appropriateness of the statistical analyses. Among the case–control studies, lower scores were mainly related to the comparability of cases and controls and the consistency of exposure measurement between the two groups. Higher scores were observed for the identification and control of confounding factors, the validity and reliability of outcome measurements, and the appropriateness of the statistical analyses (see Table S1 for details).

**Table 3:** Methodological quality assessment scores of included studies (*n* = 21).

Source	Design	Quality assessment score
Jenabi et al., 2025	cross-sectional study	7/8
Fernandes et al., 2023	cross-sectional study	6/8
Naumova et al., 2021	cross-sectional study	8/8
Pasha et al., 2021	cross-sectional study	7/8
Ho et al., 2020	cross-sectional study	5/8
Shahraki et al., 2019	cross-sectional study	5/8
Coward et al., 2019	cross-sectional study	8/8
Cao et al., 2019	cross-sectional study	7/8
Ozturk et al., 2019	cross-sectional study	5/8
Purcell-Lévesque et al., 2019	cross-sectional study	7/8
Shahraki et al., 2018	cross-sectional study	6/8
Salomão et al., 2018	case-control study	5/10
Sahin et al., 2017	cross-sectional study	8/8
Kucur Suna et al., 2016	cross-sectional study	7/8
Czyżkowska et al., 2016	cross-sectional study	8/8
Yangin et al., 2016	cross-sectional study	6/8
Gao et al., 2013	cross-sectional study	8/8
Pakpour et al., 2012	cross-sectional study	8/8
Furukawa et al., 2012	case-control study	8/10
Lotti et al., 2012	cross-sectional study	7/8
Nelson et al., 2008	cross-sectional study	5/8

Across the included studies, recurring risks of bias were related to participant selection, self-reported outcome assessment, and residual confounding. Most participants were recruited from fertility clinics or treatment centers, which may have limited the representativeness and generalizability of the study populations. Although validated questionnaires were generally used, sexual and psychological outcomes were predominantly self-reported and may therefore have been affected by recall or social desirability bias. Potential demographic, clinical, treatment-related, and psychosocial confounders were not consistently identified or addressed. In the case–control studies, differences in participant recruitment and data-collection procedures may also have affected the comparability of cases and controls.

### 3.4 Sexual dysfunction: a multifactorial and prevalent issue in patients with infertility

Thirteen of included studies reported the prevalence of sexual function problems among participants with infertility. Among women, the prevalence of sexual dysfunction ranged from 14.8% (Purcell-Lévesque et al., 2019) to 90% (Czyżkowska et al., 2016). Among men, the prevalence ranged from 6.7% (Purcell-Lévesque et al., 2019) to 61.8% (Yangin et al., 2016). These wide ranges indicate substantial heterogeneity in the reported prevalence of sexual dysfunction across the included studies, particularly among women. The included studies predominantly used validated instruments to assess sexual function. The Female Sexual Function Index (FSFI) is the most common tool for women, while the International Index of Erectile Function (IIEF) and the Premature Ejaculation Diagnostic Tool (PEDT) are standard for men. Some studies also incorporated objective measures, including scrotal B-ultrasound (Lotti et al., 2012), sperm, and testosterone levels (Coward et al., 2019). Across the included studies, poorer sexual function was also associated with several psychological and sociodemographic characteristics, including age, psychological, occupation, economic status, education level, and other socio-demographic characteristics (Furukawa et al., 2012; Pasha et al., 2017, 2020).

### 3.5 Associations of anxiety and depression with sexual function

Higher levels of anxiety and depression were significantly associated with poorer sexual function among patients with infertility. Among the 14 studies involving women with infertility, anxiety and depression were associated with decreased libido (Nelson et al., 2008; Pakpour et al., 2012; Kucur Suna et al., 2016; Shahraki et al., 2018; Ozturk et al., 2019; Shahraki et al., 2019; Ho et al., 2020; Naumova et al., 2021; Pasha et al., 2021; Fernandes et al., 2023; Jenabi et al., 2025), decreased sexual arousal (Nelson et al., 2008; Pakpour et al., 2012; Kucur Suna et al., 2016; Salomão et al., 2018; Shahraki et al., 2018, 2019; Ho et al., 2020; Naumova et al., 2021; Pasha et al., 2021; Fernandes et al., 2023; Jenabi et al., 2025), difficulty experiencing lubrication (Nelson et al., 2008; Pakpour et al., 2012; Kucur Suna et al., 2016; Shahraki et al., 2018; Purcell-Lévesque et al., 2019; Shahraki et al., 2019; Ho et al., 2020; Naumova et al., 2021; Pasha et al., 2021; Fernandes et al., 2023; Jenabi et al., 2025) and difficulty experiencing orgasm (Nelson et al., 2008; Pakpour et al., 2012; Czyżkowska et al., 2016; Kucur Suna et al., 2016; Yangin et al., 2016; Shahraki et al., 2018, 2019; Ho et al., 2020; Naumova et al., 2021; Pasha et al., 2021; Fernandes et al., 2023; Jenabi et al., 2025), and increased sexual pain (Nelson et al., 2008; Pakpour et al., 2012; Kucur Suna et al., 2016; Shahraki et al., 2018, 2019; Ho et al., 2020; Naumova et al., 2021; Pasha et al., 2021; Fernandes et al., 2023; Jenabi et al., 2025). Overall, associations were reported across multiple domains of female sexual function, particularly sexual desire, arousal, lubrication, and orgasm.

Among the 10 studies involving men with infertility, anxiety and depression were associated with lower libido (Ho et al., 2020; Fernandes et al., 2023), difficulty experiencing orgasm (Lin et al., 2012; Purcell-Lévesque et al., 2019; Ho et al., 2020; Fernandes et al., 2023), prospermia (Lotti et al., 2012; Gao et al., 2013; Cao et al., 2019; Ho et al., 2020) and impaired erectile function (Lotti et al., 2012; Gao et al., 2013; Sahin et al., 2017; Cao et al., 2019; Coward et al., 2019; Purcell-Lévesque et al., 2019; Ho et al., 2020; Fernandes et al., 2023). Overall, the findings in men mainly concerned sexual desire, erectile function, ejaculatory function, and orgasmic function.

### 3.6 Associations of anxiety and depression with intimacy

Higher levels of anxiety and depression were also associated with poorer intimacy-related outcomes among patients with infertility. Among 15 studies involving women with infertility, anxiety and depression were most commonly associated with poorer dyadic functioning (Czyżkowska et al., 2016; Ho et al., 2020), decreased marital satisfaction (Nelson et al., 2008; Yangin et al., 2016; Fernandes et al., 2023), reduced emotional expression and emotional involvement (Czyżkowska et al., 2016; Purcell-Lévesque et al., 2019), poor communication quality (Czyżkowska et al., 2016; Ho et al., 2020), and lower quality of sexual interaction between partners (Pakpour et al., 2012; Shahraki et al., 2018; Jenabi et al., 2025). Among eight surveys involving men with infertility, anxiety and depression were similarly associated with decreased relationship satisfaction (Nelson et al., 2008; Sahin et al., 2017; Cao et al., 2019), increased emotional distancing (Purcell-Lévesque et al., 2019), and cross-negative effects within couples, specifically that male attachment avoidance was associated with greater orgasmic difficulties in their female partners (Purcell-Lévesque et al., 2019). Overall, the findings suggest that anxiety and depression were associated with difficulties across the emotional, communicative, sexual, and relationship-related dimensions of intimacy.

## 4 Discussion

This systematic review included 21 studies from 11 countries, involving a total of 6,837 participants, and synthesized evidence on the associations of anxiety and depression with sexual health among individuals with infertility. Overall, sexual dysfunction was frequently reported among individuals with infertility and appeared to be multifactorial in nature. Notably, anxiety and depression were significantly associated with multidimensional impairments in sexual function in both male and female patients. Furthermore, psychological distress was associated with intimate relationships, including reduced relationship satisfaction, weakened emotional bonding, and impaired communication quality. Across studies, the associations in women extended across desire, arousal, lubrication, orgasm, pain, satisfaction, and sexual distress, whereas findings in men mainly concerned libido, erectile function, ejaculation, and orgasmic function. These findings indicate that psychological distress is associated with both individual sexual responses and broader couple-level relationship functioning.

Depression may interfere with the neurotransmitter systems in the central nervous system responsible for processing sexual information and regulating sexual responses, thereby reducing sexual motivation and pleasurable experiences (Basson and Gilks, 2018), leading to decreased sexual desire and lower sexual satisfaction. Meanwhile, under chronic stress, sustained activation of the hypothalamic–pituitary–adrenal (HPA) axis results in persistently elevated cortisol levels, exerting neurotoxic effects on the hippocampus (Sharan and Vellapandian, 2024; Lei et al., 2025). These processes may reduce neurogenesis, impairs synaptic plasticity, and disrupts cognitive and emotional regulatory functions, potentially interfering with normal sexual response processes. Previous studies have also indicated that the extent of hippocampal atrophy in depression is closely associated with both plasma cortisol levels and the duration of depressive episodes, and that such structural changes are partially reversible with remission of depressive symptoms (Sapolsky, 2000). Infertility-related anxiety may also be associated with alterations in spermatogenesis and testosterone production in men, with decreased testosterone levels being closely linked to erectile dysfunction (Coward et al., 2019). Furthermore, during sexual stimulation, persistently elevated heart rate and excessive sympathetic nervous system activation can disrupt normal sexual response rhythms, increasing the risk of premature ejaculation and attenuating penile engorgement responses (Rowland, 2005).

Patients with infertility commonly experience concerns about reproductive failure, sexual performance anxiety, and fears regarding the stability of their intimate relationships (Dube et al., 2023; Navid et al., 2023). These negative cognitions can lead to attentional distraction, excessive self-monitoring, and heightened bodily vigilance during sexual activity, thereby impairing the individual's ability to focus on sexual stimuli and physical sensations. Existing studies have shown that both men and women with higher levels of anxiety find it more difficult to relax and connect with their bodily experiences during sexual intercourse, which may further affect women's lubrication capacity as well as men's erectile and orgasmic functions (Brassard et al., 2007). Moreover, “task-oriented sex” aimed at conception reduces the spontaneity and erotic components of sexual activity and is associated with a higher prevalence of sexual dysfunction (Czyżkowska et al., 2016). Among women, such pressured and compulsory sexual contexts can also lead to increased dyspareunia (Dunkley et al., 2016) and reduced orgasm frequency (Cohen and Belsky, 2008).

Women with infertility may experience anxiety, low self-esteem, distorted perception of body image, and fear of rejection, along with feelings of shame and social pressure (Thapa et al., 2021; Jannink et al., 2024). These factors have been associated with lower sexual confidence and initiative and with a higher prevalence of sexual dysfunction. Furthermore, the available evidence suggests a possible bidirectional association between sexual dysfunction and anxiety and depression: sexual dysfunction is associated with greater anxiety and depressive symptoms, while greater anxiety and depressive symptoms are associated with sexual dysfunction (Gallinelli et al., 2001; Mezones-Holguin et al., 2011).

Anxiety and depression were associated not only with poorer individual sexual function but also with poorer partner intimacy. Symptoms such as lower interest, low energy, low self-esteem, and anhedonia may co-occur with lower relationship quality and poorer communication between partners (Ramezanzadeh et al., 2004; Furukawa et al., 2012; Czyżkowska et al., 2016).

Sexual behavior is not only an important component of marital relationships but also profoundly affects individuals' intimate experiences, self-identity, psychological health, and overall quality of life. During the diagnosis and treatment of infertility, the persistent accumulation of anxiety and depressive symptoms often affects couples' sexual life and emotional bonds. Sexual behavior, which originally serves functions of intimacy, support, and emotional soothing, may gradually transform into an activity associated with stress and feelings of failure (Fernandes et al., 2023). Furthermore, effective communication between partners is crucial for sexual relationships (Mallory et al., 2019). Previous studies have shown that improving sexual communication helps buffer the adverse effects of anxiety on sexual function, particularly among couples engaged in conception attempts (Mallory et al., 2019; Schmidt et al., 2005).

For women, this impact may be even more pronounced. Sexuality involves not only physical responses but also factors related to fertility, body image, and sociocultural expectations (Jannink et al., 2024; Ouahid et al., 2025). In many cultural contexts, women often experience higher levels of infertility-related stress, and infertility is more likely to trigger feelings of shame, stigma, and reduced self-worth (Mateane et al., 2026). Traditional beliefs about childbearing and gender roles may lead women to be viewed as primarily responsible for infertility, potentially affecting their sexual self-esteem, communication with their partners, and psychological wellbeing. In contexts where sexual matters are considered highly private, some individuals may also be less willing to discuss sexual difficulties with their partners, healthcare providers, or researchers, which may contribute to the underrecognition of sexual health problems and delays in seeking professional help. These cultural differences, however, should be interpreted cautiously, as the included studies did not use standardized cross-cultural comparative designs. In addition, infertility-related diagnostic and treatment procedures are generally more invasive and burdensome for women (Khorasani et al., 2025), which may further intensify psychological distress and negatively affect intimate relationships.

Notably, the associations between anxiety and depression and intimate relationships may involve partner interdependence. Psychological distress in one partner may be transmitted to the other through daily interactions, leading to a synchronous deterioration in both partners' emotional states and weakening emotional expression, communication quality, and the ability to cope with stress together (Bodenmann et al., 2007; Ho et al., 2020; Purcell-Lévesque et al., 2019; Cao et al., 2019). Psychological factors were significantly associated with sexual problems, sexual difficulties, and sexual dysfunction, highlighting the need for a more comprehensive approach to understanding and addressing sexual health in the context of infertility. However, because relatively few included studies assessed both partners or used dyadic analytical approaches, evidence regarding partner interdependence and cross-partner associations remains limited.

Accordingly, the consistent associations identified in this review support consideration of routine screening for anxiety and depression as part of infertility diagnosis and treatment to enable early identification of at-risk individuals and timely intervention. At the same time, sexual health assessment should be considered an important component of standardized infertility care rather than being marginalized (Esteves, 2026). Given the interplay among infertility-related psychological distress, sexual dysfunction, and impaired intimate relationships, a multidisciplinary comprehensive intervention model may be beneficial, in which psychological interventions may alleviate emotional symptoms and sex therapy may improve sexual function and intimate relationship quality. Of particular note, interventions should be implemented more at the couple level rather than at the individual level to enhance partner communication, strengthen emotional support, and promote mutual understanding (Gorman et al., 2022). Future efforts should further establish interdisciplinary collaboration mechanisms among reproductive physicians, psychotherapists, and sex therapists, effectively integrating mental health services into routine reproductive medical care to improve the overall health outcomes and quality of life of patients with infertility.

### 4.1 Limitations

This review has several limitations. First, only studies published in Chinese or English were included, which may have introduced language bias. In addition, gray literature, such as dissertations and conference abstracts or proceedings, was excluded according to the eligibility criteria, potentially increasing the risk of publication bias. Second, most of the included studies were cross-sectional, which allowed the identification of associations but precluded the establishment of temporality or causal relationships. Reverse causation therefore cannot be excluded, and further research is required to elucidate the underlying mechanisms. Third, substantial heterogeneity in the instruments used to assess anxiety, depression, and sexual function precluded meta-analysis and limited direct comparisons across studies and between women and men. The included studies also did not consistently report findings according to primary vs. secondary infertility, infertility etiology, or cultural context, preventing reliable subgroup and cross-cultural comparisons. Fourth, sexual and psychological outcomes were predominantly self-reported, which may have introduced recall and social desirability bias. Given the sensitive nature of these topics, some participants may also have been reluctant to respond or may have underreported symptoms. Fifth, none of the included studies systematically screened for or excluded pre-existing anxiety or depressive disorders prior to infertility diagnosis, making it impossible to determine whether the observed psychological distress was attributable to infertility itself or to pre-existing conditions. In addition, potential demographic, clinical, treatment-related, and psychosocial confounders were not consistently identified or adjusted for across studies, and residual confounding therefore cannot be excluded. Finally, most participants were recruited from fertility clinics or treatment centers, which may also have limited the representativeness and generalizability of the findings. Taken together, the overall confidence in the available evidence is limited because of the predominantly observational and cross-sectional study designs, methodological limitations, potential sources of bias, residual confounding, and substantial heterogeneity in measurement approaches and study methods. Therefore, although the available evidence generally suggests associations of anxiety and depression with sexual health among infertility patients, the findings should be interpreted cautiously, particularly with regard to the magnitude and causal nature of these associations. Addressing these limitations represents a crucial direction for future research endeavors.

## 5 Conclusions

Anxiety and depression were associated with poorer sexual health outcomes among patients with infertility, especially in terms of spousal intimate relationships and sexual function. These findings support consideration of psychological counseling and sexual health screening as components of comprehensive infertility care. Such screening may be conducted by trained fertility nurses or reproductive medicine physicians at the initial assessment and repeated when clinically indicated, using validated instruments, with referral to mental health professionals, sexual health specialists, or couple-based counseling services as needed. Family and partner support may also be considered; however, the effectiveness of such support in improving patients' physical and psychological wellbeing and relationship outcomes remains to be established through prospective or interventional studies.
