# Supplementary material for: Associations of anxiety and depression with sexual health among infertility patients: a systematic review
Source: Front Psychol. 2026 Aug 28;17:1823084. doi: 10.3389/fpsyg.2026.1823084 (PMC13536997; doi:10.3389/fpsyg.2026.1823084)
Supplement: Supplementary file 1 [file Table_1.docx]

Table S1 Methodological quality assessment of the included studies

| **cross-sectional study** | 1. Were the criteria for inclusion in the sample clearly defined? | 2. Were objective, standard criteria used for measurement of the condition? | 3. Was the exposure measured in a valid and reliable way? | 4. Were the outcomes measured in a valid and reliable way? | 5. Were confounding factors identified? | 6. Were strategies to deal with confounding factors stated? | 7. Was appropriate statistical analysis used? | 8. Were the study subjects and the setting described in detail? |  |  |
| --- | --- | --- | --- | --- | --- | --- | --- | --- | --- | --- |
| Jenabi et al., 2025 | Y | Y | Y | Y | Y | N | Y | Y |  |  |
| Fernandes et al., 2023 | Y | N | Y | Y | Y | N | Y | Y |  |  |
| Naumova et al., 2021 | Y | Y | Y | Y | Y | Y | Y | Y |  |  |
| Pasha et al., 2021 | Y | U | Y | Y | Y | Y | Y | Y |  |  |
| Ho et al., 2020 | Y | N | Y | Y | N | N | Y | Y |  |  |
| Shahraki et al., 2019 | Y | U | Y | Y | U | U | Y | Y |  |  |
| Coward et al., 2019 | Y | Y | Y | Y | Y | Y | Y | Y |  |  |
| Cao et al., 2019 | Y | U | Y | Y | Y | Y | Y | Y |  |  |
| Ozturk et al., 2019 | Y | U | Y | Y | N | N | Y | Y |  |  |
| Purcell-Lévesque et al., 2019 | Y | U | Y | Y | Y | Y | Y | Y |  |  |
| Shahraki et al., 2018 | Y | U | Y | Y | Y | Y | Y | N |  |  |
| Sahin et al., 2017 | Y | Y | Y | Y | Y | Y | Y | Y |  |  |
| Kucur Suna et al., 2016 | Y | Y | Y | Y | Y | N | Y | Y |  |  |
| Czyżkowska et al., 2016 | Y | Y | Y | Y | Y | Y | Y | Y |  |  |
| Yangin et al., 2016 | Y | N | Y | Y | Y | N | Y | Y |  |  |
| Gao et al., 2013 | Y | Y | Y | Y | Y | Y | Y | Y |  |  |
| Pakpour et al., 2012 | Y | Y | Y | Y | Y | Y | Y | Y |  |  |
| Lotti et al., 2012 | Y | Y | Y | Y | Y | Y | Y | N |  |  |
| Nelson et al., 2008 | N | N | Y | Y | Y | Y | Y | N |  |  |
| **case-control study** | 1. Were the groups comparable other than the presence of disease in cases or the absence of disease in controls? | 2. Were cases and controls matched appropriately? | 3. Were the same criteria used for identification of cases and controls? | 4. Was exposure measured in a standard, valid and reliable way? | 5. Was exposure measured in the same way for cases and controls? | 6. Were confounding factors identified? | 7. Were strategies to deal with confounding factors stated? | 8. Were outcomes assessed in a standard, valid and reliable way for cases and controls? | 9. Was the exposure period of interest long enough to be meaningful? | 10. Was appropriate statistical analysis used? |
| Salomão et al., 2018 | N | N | Y | U | N | Y | Y | Y | U | Y |
| Furukawa et al., 2012 | N | Y | Y | Y | U | Y | Y | Y | Y | Y |

Y, Yes; N, No; U, Unclear.
